# Injectable hydrogels for bone regeneration: mechanical reinforcement strategies using nanoparticles and nanofibers

**DOI:** 10.5599/admet.3037

**Published:** 2026-01-03

**Authors:** Morteza Mirzagoli, Fariba Ganji, Lobat Tayebi

**Affiliations:** 1 Biomedical Engineering Group, Faculty of Chemical Engineering, Tarbiat Modares University, Tehran, Iran, 14115-143, Iran; 2 Institute for Engineering in Medicine, Health, & Human Performance (EnMed), Batten College of Engineering and Technology, Old Dominion University, Norfolk, VA, 23529, USA

**Keywords:** Bone tissue engineering, *in situ* forming scaffold, nanomaterials

## Abstract

**Background and Purpose:**

The growing demand for bone regeneration following severe injuries highlights the importance of scaffolds in bone tissue engineering (BTE). Injectable hydrogels have emerged as promising candidates because their properties closely mimic the native extracellular matrix (ECM). However, their limited mechanical strength and structural instability restrict their practical application.

**Approach:**

This review summarizes recent strategies for reinforcing in situ-forming injectable hydrogels to improve their mechanical performance for bone regeneration. Particular emphasis is placed on nanomaterial-based strategies, including the incorporation of nanoparticles and nanofibers, and their ability to enhance the physical properties of polymeric networks.

**Key Results:**

Evidence from recent studies demonstrates that reinforcing hydrogels with nano-scaled materials creates interconnected networks that improve load-bearing capacity, stability, and resistance to deformation. These reinforced systems retain the inherent advantages of injectable hydrogels-biocompatibility, biodegradability, permeability to oxygen and nutrients, and drug delivery capability-while addressing their mechanical shortcomings.

**Conclusion:**

Nanomaterial-based reinforcement offers a versatile approach to overcoming the limitations of injectable hydrogels in BTE. By providing improved structural integrity alongside biological functionality, these advanced systems broaden the potential of injectable hydrogels for clinical translation. Future work should focus on optimizing reinforcement strategies to balance mechanical enhancement with safety, manufacturability, and regulatory considerations.

## Introduction

Bone is a mineralized connective tissue essential for structural support, organ protection, and mineral storage [1]. It consists of various cell types, including osteoblasts, osteocytes, osteogenic cells, and osteoclasts [2]. Osteoblasts are responsible for synthesizing type I collagen, the main component of the bone matrix, while osteocytes represent the most abundant bone cells [3]. Osteogenic cells, derived from mesenchymal stem cells (MSCs), play a crucial role in bone remodelling and regeneration [4]. Osteoclasts, which originate from the monocyte-macrophage lineage, are involved in bone resorption and contribute to bone turnover [5]. Bones are classified into two types: cortical and trabecular. Cortical bone constitutes approximately 90 % of the skeleton, provides mechanical strength, and has a porosity of 3.5 % [6,7]. In contrast, trabecular bone, found at the ends of long bones and within vertebrae, exhibits higher porosity (~79.3 %) and metabolic activity [7,8].

These properties vary with age and anatomical location [11]. Cortical bone is anisotropic, exhibiting greater mechanical strength along its longitudinal axis and increased brittleness at high strain rates. The longitudinal direction, which aligns with the diaphyseal axis, demonstrates higher strength and greater tensile/compressive moduli compared to the radial and circumferential directions (Table 1). As shown in Table 2, trabecular bone exhibits superior compressive behaviour, yielding at 0.7 % strain and tolerating up to 50 % strain. Excessive loading and unloading may cause permanent deformation or fractures [9].

**Table 1.** Mechanical properties of cortical bone in longitudinal and transverse directions [9,10].

Property	Longitudinal direction	Transverse direction
Elastic modulus, MPa	17900 ± 3900	10100 ± 2400
Poisson's ratio	0.62 ± 0.26	0.62 ± 0.26
Compressive yield strain, %	0.98 ± 0.09	0.83 ± 0.42
Compressive yield stress, MPa	115.06 ± 16.3	41.8 ± 19.4
Compressive ultimate stress, MPa	205 ± 17.3	131± 20.7
Tensile ultimate stress, MPa	0.67 ± 0.04	53 ± 10.7
Storage modulus., GPa	17.1 ± 0.8	26.7± 1.5
Loss modulus, GPa	0.40 ± 0.01	0.47 ± 0.03

**Table 2.** Mechanical properties of trabecular bone

Property	Value	Reference
Elastic modulus (GPa)	13.0 ± 1.47	[12]
Young's modulus (GPa)	10.4 ± 3.5	[13]
Tensile yield stress (MPa)	0.78 ± 0.04	[14]
Compressive yield strain (MPa)	0.84 ± 0.06	
Poisson's ratio	0.3	[15]
Storage modulus (GPa)	13.75 ± 3.21	[16]
Loss modulus (GPa)	6.39 ± 1.28	[16]

**Figure 1.**
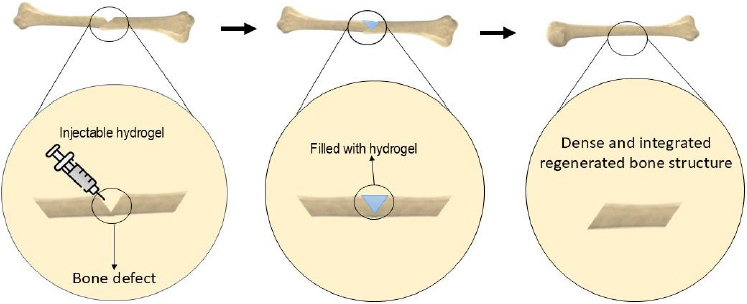
Formation and application of injectable hydrogels for treating bone defects.

Extensive bone loss due to trauma, tumours, or genetic conditions often exceeds the body's natural regenerative capabilities [17]. Tissue engineering (TE) has emerged as an innovative strategy to address this issue [18]. Bone tissue engineering (BTE) integrates stem cells, signalling molecules, and scaffolds to facilitate bone regeneration [19]. Among these, scaffolds are essential components that replicate the structure and function of native ECM, thereby promoting cell adhesion, proliferation, and differentiation. Ideal scaffolds must be biodegradable, biocompatible, osteoconductive, osteoinductive, and bioactive [20]. Among biomaterials, hydrogels are widely used in BTE owing to their favourable biological and mechanical properties [21]. They can be used as pre-formed implants or as injectable materials, which transition from liquid to solid state post-injection [22]. Injectable hydrogels offer a minimally invasive approach compared with conventional surgical implantation, enabling the material to adapt precisely to the defect shape (Figure 1) [23,24].

Despite these benefits, the low mechanical integrity of injectable hydrogels remains a critical challenge [25,26]. Furthermore, mechanical cues in the cellular microenvironment significantly influence stem cell fate. MSCs differentiate into neurons on soft substrates (0.1 to 1.0 kPa), into muscle cells on moderately stiff substrates (8 to 17 kPa), and into osteoblasts on stiffer matrices (34 kPa) [27,28]. Similar trends are observed in 3D hydrogels: osteogenesis is promoted at stiffness levels between 11 to 30 kPa, while adipogenesis occurs at 2.5 to 5.0 kPa [29]. Consequently, improving the mechanical properties of injectable hydrogels to match native bone tissue remains a major objective in BTE.

**Figure 2.**
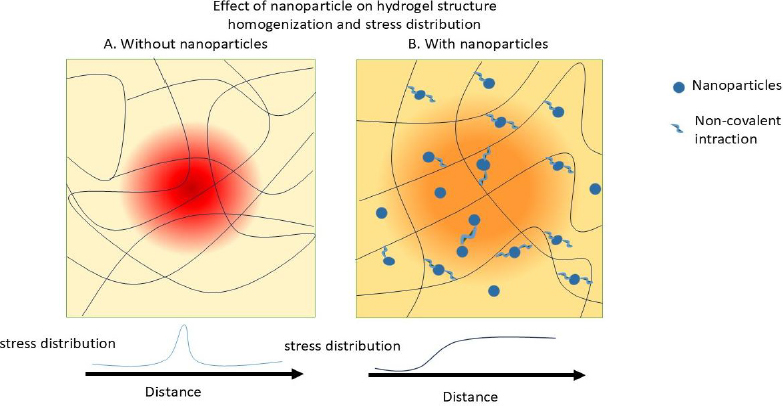
Mechanisms of reinforcement and interactions between nanomaterials and polymeric matrices

To address this, researchers have employed various strategies, including increasing polymer concentration, developing double- or interpenetrating networks, and reinforcing hydrogels with nanoparticles (NPs) and nanofibers [30]. Figure 2 schematically illustrates how NPs enhance the mechanical properties of hydrogels.

**Figure 3.**
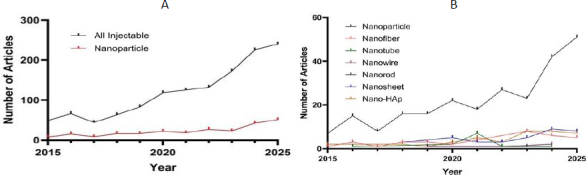
(A) number of published articles in the field of bone tissue engineering involving injectable hydrogels between 2015 and 2025, as indexed in Scopus, with the subset of studies incorporating NPs highlighted; (B) distribution of these publications according to the types of NPs used during the same period

Figure 3A illustrates the number of published articles in the field of bone tissue engineering involving injectable hydrogels between 2015 and 2025, as indexed in Scopus. The subset of studies incorporating NPs is also highlighted. Figure 3B presents the distribution of these publications according to the types of NPs used during the same period.

This review emphasizes recent advances in reinforcing in situ-forming injectable hydrogels with nanostructured additives. These include bioceramic NPs like hydroxyapatite (nHAp), calcium phosphate (CP), bioactive glass, and whitlockite; nanotubes and nanofibers such as halloysite nanotubes (HNT), carbon nanotubes (CNT), and cellulose nanofibers; as well as strategies involving ionic doping (e.g., strontium) and graphene oxide-based nanocomposites. The effectiveness of each method in enhancing the mechanical characteristics of hydrogels is critically discussed.

## Mechanical enhancement of injectable hydrogels by NPs

The incorporation of nanoscale structures into polymeric matrices yields nanocomposite hydrogels with superior mechanical properties. Table 3 summarizes recent nanocomposite hydrogels developed to address limitations in mechanical performance and osteoconductivity associated with conventional injectable hydrogels. Materials such as gelatine, dextran, alginate, chitosan, and hyaluronic acid have frequently been used as hydrogel matrices in bone tissue engineering. The use of various nanomaterials as reinforcing agents significantly improves their performance. This section explores the effects of these nanostructures on hydrogel mechanics and highlights opportunities for future innovation in the field.

**Table 3.** Injectable hydrogels applied for BTE

Main hydrogel composition	Fillers	Hydrogel crosslinker	Mechanical properties	Scaffold properties	Ref.
Gelatin/Dextran	Polydopamine/nHAp	Schiff base crosslinking	Compressive strength of 155 to 228 kPa.	Self-healing, adhesive, antioxidant, osteo-inductive	[31]
Alginate	Alendronate	Ionic gelation	Compressive strength of 0.48 to 7.16 kPa	Injectable, osteogenic, biocompatible, biodegradable, flexible, drug release scaffold	[24]
Chitosan	Black phosphorus nanosheets	β-Glycerol phosphate	-	Excellent photothermal effects, controlled release scaffold with high drug-loading capacity, biocompatible, osteogenic	[32]
Collagen/Hyaluronic acid	Calcium sulphate nanorods	Bio-orthogonal click reaction	Compressive strength of 6.3 to 16 Kpa.	Enhance cell adhesion and proliferation, self-biomineralization, stimulated the preosteoblasts differentiation	[33]
Alginate	nHAp / Laponite NPs	Ionic gelation	Maximum compressive stresses of 50 kPa.	Biocompatibility, bone-inducing activity, tuneable physical behaviours	[34]
Alginate/silk fibroin	----	Ionic gelation and Schiff-base reaction	Compressive modulus of 67 kPa	Dual-crosslinked, high cell-scaffold interactions	[35]
Alginate/gelatin	Carbone Nanofiber	Schiff base reactions	Compressive strengths of 63 to 102 Kpa.	High compressive modulus, self-healing, good biomineralization, enhanced preosteoblast cell viability, proliferation, osteogenic	[36]
GelMA / Hyaluronic acid methacrylate	nHAp- exosomes	Photo-crosslinking	Compressive moduli of 8.4 to 21 kPa.	Controlled release scaffold, promote osteogenesis and formation of H-type vessels	[37]
Silk fibroin/ sodium alginate	Mesoporous bioglass	Ionic gelation	Compressive strength of ~0.5 MPa.	Self-adaptability in mechanical reinforcement and degradation, induce new bone formation and angiogenesis	[38]
DNA/dexamethasone (Dex)	Dex-loaded HNTs	Physical crosslinking	Storage modulus of 100 to 200 Pa for 3 wt.% HNTs	Eliminates chemical modifications, high biocompatibility & osteogenic activity, controlled drug release	[39]
Gelatine/alginate	LAPONITE^®^ XLG nanosilicates	Ionic gelation	LAP significantly increased the viscosity	Mimics natural ECM, preserves survival and functionality of laden cells during injection	[40]
Oxidized chondroitin sulphate /gelatine (gel)	Amino-modified barium titanate (KBTO) NPs	Dynamic covalent crosslinking through Schiff base and hydrogen bonds	Storage modulus increases from 2160 to 2560 Pa for 0.5 wt.% KBTO NPs	Bone-adhesive, electrically accelerated bone healing	[41]
Gelatine	Tannic acid-mineral NPs	Physical crosslinking	Storage modulus increases from 6990 to 8030 Pa for 1 mg ml^-1^ tannic acid-mineral NPs	Biocompatibility, ion release profile	[42]
GelMA / alginate-graft-dopamine	Copper-decorated MXene nanosheets	Photo-polymerization and Ionic gelation	Storage modulus increases from 250 to 1250 Pa for 100 μg mL^-1^ MXene	Antibacterial and antioxidant properties, osteogenesis and angiogenesis, enhanced mechanical properties, adhesion, self-healing	[43]
Pluronic F127 (PF-127)	Calcium peroxide embedded PCL microspheres and nHAp/Laponite NPs	Physical crosslinking	Storage modulus of 10 kPa, with 100 μg/mL of nHAp and 2 % wt Laponite	Sustained oxygen release, enhanced osteoinductivity, promotion of vascularization and bone regeneration	[44]
GelMA / κ-carrageenan	Calcium phosphate cements (CPC)	Photo-crosslinking	Storage modulus increases from 10 to over 100 kPa for 10 wt.% CPC	Osteogenic activity and bioactivity, rapid gelation, enhanced mechanical strength	[45]
Silk Fibroin/aldehyde-cellulose nanocrystalline	Polyetheretherketone	Schiff base reaction	1.1-fold higher magnitude of Storage modulus	Osteogenic induction, cytocompatibility, enhanced bone regeneration	[46]
Carboxymethyl chitosan/oxidized dextran	nHAp and strontium NPs modified with zoledronic acid	Schiff base reaction	High storage modulus of 1400 Pa	Osteogenic, bioactivity, antibacterial activity	[47]
GelMA	Nanotricalcium phosphate@polydopamine NPs modified with QK peptide	Photo-crosslinking	High maximum stress	Biocompatibility, osteogenic and bone regeneration, pH-responsive release	[48]
Phenylboronic acid modified gelatine/oxidized-dextran	Lithium and cobalt co-doped mesoporous bioactive glass NPs (MBGNs)	Schiff base reaction	Compressive modulus increases from 25 to 90 kPa for 5 wt.% MBGNs	Good cytocompatibility, osteogenic and angiogenic, sustained release	[49]
Regenerated silk fibroin	MXene nanosheets	Enzymatic and physical Crosslinking	MXene did not significantly affect the compressive or elastic modulus	Promotion of osteogenesis, electroactivity and conductivity, biocompatibility, promotion of angiogenesis	[50]
GelMA	Silver-doped nHAp and MXene	Photo-crosslinking	Maximum compressive stress of 151 kPa	Antibacterial activity, osteoinductive and osteogenic capabilities, biocompatibility	[51]
Dopamine-modified gelatine	nHAp and polydopamine functionalized nHAp	-	Compressive strength of 100 to 500 kPa	Cell adhesion, proliferation, excellent biocompatibility, accelerate bone repair	[52]

### Bioceramic nanoparticles

Bioceramics and inorganic NPs enhance the mechanical performance of hydrogels through multiple complementary mechanisms.

Primarily, these nanofillers can interact with the hydrogel network, particularly with polymer chains containing some functional groups. Such interactions, mainly hydrogen bonding or other weak physical forces, increase the crosslinking density within the polymer matrix. Moreover, the abundant active sites on the surface of NPs, such as hydroxyapatite, enable them to function as multifunctional crosslinking nodes, thereby further reinforcing the hydrogel network.

In addition, these inorganic particles possess a substantially higher elastic modulus than polymeric matrices. When homogeneously dispersed within the hydrogel, they facilitate efficient load transfer between the soft polymeric phase and the rigid inorganic domains, preventing localized deformation and microcrack propagation. As a result, the composite hydrogels exhibit improved mechanical strength and are particularly suitable for load-bearing applications.

Furthermore, these NPs can act as nucleation centres for calcium and phosphate deposition or mineral crystal growth within the hydrogel. This *in situ* mineralization process leads to the formation of a local mineral phase over time, thereby enhancing the stiffness, toughness, and structural stability of the composite hydrogel.

### Hydroxyapatite nanoparticles

Hydroxyapatite (HA), chemically known as Ca_10_(PO_4_)_6_(OH)_2_, is the predominant inorganic component of natural bone. Its nanoscale form, nHAp, offers superior bioactivity and enhanced bone integration compared to porous HA [53,54]. Moreover, nHAp has demonstrated the ability to promote the osteogenic differentiation of mesenchymal stem cells (MSCs) [55]. Table 4 presents recent studies utilizing nHAp to reinforce injectable hydrogels. These studies reveal that physical incorporation of nHAp into polymer matrices significantly improves mechanical strength and modulus.

**Table 4.** Mechanical reinforcement of injectable hydrogels using nHAp

Hydrogel composite	Form and content of nHAp used	Dispersion method / average size	Changes in mechanical properties	Ref.
Chitosan/ polyelectrolyte complex	Physical blending of 1 % w/v nHAp crystals	Sonication / not given	Stiffness increased from 20 to 30 kPa for 1 % w/v nHAp.	[59]
Gelatine, chitosan, polyvinyl alcohol	Physical blending of 0.75, 3, 12.5 and 50 % w/v nHAp	Fully mixed / not given	Compressive strength improved from 0.82 to 1.39 MPa for 3 to 50 % w/v nHAp	[60]
Chitin / PCL	Physical blending of 1 % w/v nHAp	Dispersed uniformly / <200 nm	Elastic modulus increased from 7.2 to 14.2 KPa for 1 % w/v nHAp.	[61]
GelMA	Physical blending of 10 mg mL^-1^ nHAp	Sonication / not given	Compressive modulus increased from 7.5 to 10 kPa	[62]
Alginate	Physical blending of 5 g needle-like nHAp crystals in 1, 2 and 4 ml of alginate solution	Sonication / 20-30 nm in diameter and 90-110 nm in length	Compressive strength decreased from 7.9 to 5.9 MPa	[63]
Chitosan / gelatine	Physical blending of 10, 20, 30, 40 and 50 % w/v nHAp crystals	Not given/ 35 to 122 nm	Compressive strength increased from 202 to 290 kPa for 20 to 50 % w/v nHAp	[64]
GelMA / methylacrylylated hyaluronic acid	Physical blending of 0.5 and 1 % w/v nHAp crystals	Strong vortexing / not given	Compressive strength nearly reached 1 MPa for 1 % w/v nHAp	[65]
Modified gelatin / hyaluronic acid	Physical blending of 3 % w/v nHAp crystals	Well dispersed / <200 nm	Storage modulus increases from 4 to 8 kPa for 3 wt.% nHAp	[66]
Pluronic grafted silk fibroin	Physical blending of 5, 10 and 20 % w/v nHAp	Sonication / spherical and polyhedral morphologies (from 25 to 45 nm)	Young's modulus reaches 0.035 MPa for 20 % w/v nHAp	[67]
Chitosan-tripolyphosphate	Physical blending of0. 5, 1 and 2 % w/v nHAp crystals	Sonication / < 200 nm particle size	Compressive strength increases from 2.5 to 3 MPa for 2 % w/v nHAp	[68]
Carboxymethyl-chitosan/gelatine	Physical blending of 40 % w/v nHAp crystals	Not given but well dispersed / hydrodynamic radius of 208 nm	Elastic modulus increases from 142 to 921 Pa for 40 % w/v nHAp	[69]

nHAp enhances the mechanical properties of hydrogels through several mechanisms. Firstly, they can interact with the hydrogel network, particularly with polymer chains containing hydroxyl, carboxyl, or phosphate functional groups. These interactions, which may involve hydrogen bonding or other weak physical forces, increase the crosslinking density within the polymer network. Moreover, due to the presence of multiple active sites on the surface of Hap NPs, they can serve as multifunctional nodes that further enhance the effective crosslinking density of the hydrogel [56].

Owing to its high elastic modulus, well-dispersed nHAp facilitates effective load transfer between the polymeric and inorganic phases, enhancing stiffness and strength. Additionally, nHAp serves as a nucleation centre for mineral deposition, promoting in situ mineralization and long-term structural stability. Its bone-mimicking composition further supports both mechanical reinforcement and favourable cellular responses [57,58].

Wasupalli *et al.* [59] developed a thermosensitive chitosan-based hydrogel incorporating nHAp and polyelectrolyte complex (PEC) fibres. When β-glycerophosphate (β-GP) was used as a crosslinker, 1 % (w/v) nHAp dispersed uniformly within the matrix, while 2 % caused aggregation. The resulting hydrogels demonstrated about a 2.5-fold increase in stiffness compared to unreinforced chitosan-β-GP gels, mainly due to the synergistic effect between the fibrous network and surface-bound nHAp crystals. These gels exhibited enhanced durability and mechanical strength without evidence of cytotoxicity, likely because the combined use of NaHCO_3_, β-GP, and HAp reduced the overall salt concentration, thereby minimizing hyperosmolality and associated cell damage.

In another study, Ma *et al.* [60] fabricated composite scaffolds from gelatine, chitosan, polyvinyl alcohol, and varying nHAp concentrations (3-50 wt.%). Mechanical testing revealed that increasing nHAp content increased compressive strength from 0.82 MPa to 1.71 MPa and the compressive modulus from 3.73 MPa. The incorporation of hydroxyapatite enhanced the scaffold’s surface bioactivity and further promoted the osteogenic differentiation of rat bone marrow stem cells (BMSCs).

### Calcium phosphate nanoparticles

Calcium phosphate (CaP) materials, composed of Ca^2+^ and various phosphate anions (*e.g.* PO_4_^3-^, PO_3_^-^), are widely used in BTE due to their biocompatibility and osteoinductive properties [70]. They facilitate osteoblast differentiation and mineralization, resembling the mineral composition of natural bone.

Similar to the mechanisms discussed for hydroxyapatite NPs, calcium phosphate NPs enhance the mechanical properties of hydrogels through interfacial bonding with polymer chains, efficient load transfer, increased crosslinking density, and in situ mineralization. Moreover, their bone-mimicking composition enhances the stiffness, strength, and biological performance of the composite hydrogel.

Nie *et al.* [71] prepared porous nanocomposite hydrogels by integrating biphasic CaP NPs (needle-like BCP-NPs) into a chitosan/gelatine matrix using a freeze-thaw method. The mixture was stirred at room temperature for 24 hours. Varying the BCP-NP content between 25 and 50 % (w/v) produced compressive strengths from 1.2 to 2.5 MPa. The enhancement in mechanical properties was attributed to the homogeneous dispersion of rod-like BCP NPs (average length ≈100±6 nm) within the chitosan/gelatine matrix, which prevented aggregation and promoted efficient stress transfer. Their elongated morphology served as reinforcing fillers, while the multi-scale porous architecture preserved both porosity and structural strength, yielding mechanical properties comparable to cancellous bone. Among the samples, S111 and S31, with the lowest BCP-NP concentration, exhibited superior cell proliferation but lower ALP activity. *In vivo* implantation further confirmed the gradual formation of new bone within the scaffolds, demonstrating that the CGB nanocomposites have strong potential as cancellous bone substitutes for bone tissue engineering.

Kim *et al.* [72] developed an injectable methylcellulose hydrogel by in situ formation of CaP NPs via addition of a precursor salt. CaP NPs with sizes of 40 to 50 nm were incorporated into the hydrogel, resulting in a notable increase in the storage modulus from below 10 kPa to over 20 kPa, indicating effective mechanical reinforcement. Cell viability assays demonstrated that MC hydrogels containing CaP NPs supported normal cell morphology and survival without inducing cytotoxic effects. Moreover, the MC-HAp NP composite hydrogel promoted significantly greater new bone formation, particularly mature bone tissue, compared to the pure MC hydrogel.

### Bioactive glasses

Bioactive glasses (BGs) are silicate-based ceramics that can form strong bonds with bone by generating hydroxyapatite layers on their surface and interacting with collagen in physiological environments. One widely used composition is Bioglass^®^ 45S5, which consists of 45 % SiO_2_, 24.5 % Na_2_O, 24.5 % CaO and 6 % P_2_O_5_. BGs exhibit excellent bioactivity and are extensively used in bone tissue engineering due to their osteoconductivity and biocompatibility [73].

BGs enhances the mechanical properties of hydrogels through several mechanisms. Firstly, BGs NPs (nBGs) possess surface silanol groups (≡Si-OH) capable of forming hydrogen bonds or ionic interactions with functional groups on hydrogel polymers (-COOH, -OH, -NH_2_). These interactions enhance interfacial adhesion and increase the effective crosslinking density, restricting polymer chain mobility and reinforcing the network [74]. Secondly, BG NPs exhibit a substantially higher elastic modulus than the hydrogel matrix. When uniformly distributed, they act as rigid nanofillers that efficiently transfer and distribute applied stress, mitigating localized deformation and crack propagation [75].

Thirdly, upon incorporation into the hydrogel, nBGs gradually release silicon ions (Si^4+^), which, in the presence of water and oxygen, can recondense to form silicate (Si-O-Si) bonds within the polymer matrix [76]. This process establishes a secondary inorganic network, creating an additional mineral scaffold that further enhances the stiffness, strength, and structural stability of the hydrogel. Fourthly, released ionic species, particularly Ca^2+^, can also act as ionic crosslinkers by bridging negatively charged sites on the hydrogel (*e.g.* -COO^-^ groups), reducing swelling and improving dimensional stability under load [77]. Moreover, the surface of nBGs promotes the formation of hydroxycarbonate apatite (HCA), generating a bone-like interface within the hydrogel that enhances mechanical cohesion [78].

Moreira *et al.* [79] developed an injectable thermoresponsive composite hydrogel by blending chitosan (2.5 % w/v) and β-glycerophosphate (16 % w/v) with gelatine (2.0 % w/v) and bioactive glass NPs (10 wt.%). AFM analysis confirmed the spherical morphology of the bioactive glass particles, with diameters ranging from 15 to 75 nm (mean ≈52 nm). Incorporation of bioactive glass increased the hydrogel’s elastic modulus from 5.4 Pa (pure chitosan) to 12.4 Pa and improved its viscoelastic behaviour and gelation kinetics. The enhanced elasticity and reduced gelation time upon addition of gelatine and BG suggest the formation of additional molecular interactions among components during gelation. These *in vitro* findings indicate that BG and gelatine create a more favourable microenvironment for cellular activity.

**Figure 4.**
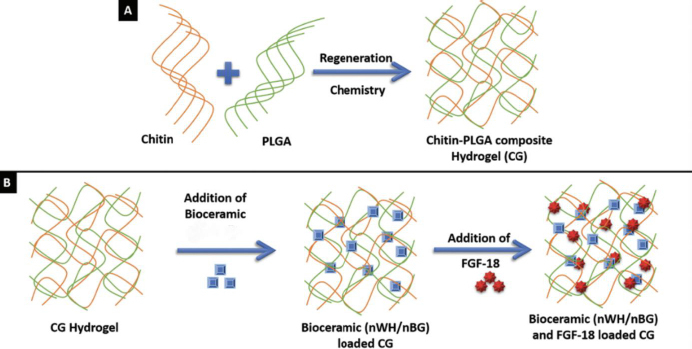
Schematic showing the preparation of chitin-PLGA hydrogel (CG) through solvent regeneration chemistry (A) and physically blending BGs or WH NPs, and finally addition fibroblast growth factor (FGF-18) to promote the osteogenic and neo-bone formation potential of final hydrogel [80] Copyright [2021] by Royal Society of Chemistry. Reprinted with permission

As shown in Figure 4, Amirthalingam *et al.* [80] synthesized an injectable chitin-poly(lactic-co-glycolic acid) (PLGA) hydrogel (CG) incorporating three types of bioceramics: nHAp, nano-whitlockite (nWH), and nano-bioactive glass (nBG). The synthesized nBG exhibited a near-spherical morphology with an average size of 14±3 nm. The CG-nBG hydrogel demonstrated the highest storage modulus (53.3±3.2 kPa), significantly exceeding those of CG-nHAp (21.5±1.6 kPa) and CG-nWH (29±5.6 kPa), indicating superior mechanical reinforcement by bioactive glass. This enhancement is attributed to the strong interaction between nBG and the polymeric matrix, as well as its smaller particle size and larger specific surface area.

Hydrogels containing nBG supported robust cell proliferation comparable to control groups and promoted the formation of capillary-like tubular structures, suggesting pro-angiogenic potential. This effect is likely due to the release of Si^4+^ ions, which stabilize HIF-1α and stimulate angiogenesis. Moreover, nBG-incorporated systems showed elevated ALP activity and upregulated osteogenic gene expression, including COL1A, confirming their superior osteogenic and mechanical performance.

### Whitlockite

Whitlockite (WH) is a magnesium-containing calcium phosphate mineral structurally similar to hydroxyapatite. It exhibits improved solubility, mechanical strength, and biological function under physiological conditions [81]. Due to its negative surface charge and magnesium content, nano-whitlockite (nWH) is regarded as a promising material for bone substitution.

Whitlockite (WH) NPs enhance the mechanical properties of hydrogels through multiple complementary mechanisms. First, they can establish noncovalent interactions with functional groups on polymer chains (-OH, -COOH, -NH_2_), thereby increasing the effective crosslinking density and reinforcing the polymer network, thereby improving hydrogel strength [82]. In addition, owing to their intrinsic hardness, WH NPs serve as rigid nanofillers that efficiently transmit and distribute applied stress throughout the hydrogel matrix, thereby enhancing mechanical performance [83]. Moreover, the Mg^2+^ and Ca^2+^ ions released from WH can serve as ionic crosslinkers, bridging negatively charged sites on the polymer (*e.g.* -COO^-^ groups), reducing swelling and improving dimensional stability under load.

Amirthalingam *et al.* [84] developed an in situ-forming injectable hydrogel composed of oxidized alginate and gelatine, reinforced with nWH particles (5 wt.%). Whitlockite NPs (80 ± 8 nm), synthesized by precipitation, enhanced the hydrogel’s mechanical performance, increasing the storage modulus from 2.1 to 3.2 kPa. This improvement is mainly attributed to ionic crosslinking, uniform dispersion, and the stiff mineral nature of WH. Additionally, WH NPs promoted cell adhesion and osteogenic activity by releasing Mg^2+^ and phosphate ions, which stimulate osteogenic differentiation through the ERK signalling pathway, upregulating RUNX2 and BMP2 expression and thereby enhancing bone regeneration potential.

In another study, Yegappan *et al.* [85] fabricated a carrageenan-based injectable hydrogel enriched with nWH and dimethyloxallyl glycine, an angiogenic drug. Whitlockite NPs (75±8.5 nm, polygonal) were incorporated into the precursor solution to form a nanocomposite hydrogel. Rheological analysis revealed an increase in storage modulus from 282.1±5.6 to 371±7.7 Pa, confirming the mechanical reinforcement effect of the ceramic fillers. The hydrogel exhibited excellent biocompatibility, supporting endothelial cell migration and capillary-like structure formation, with DMOG serving as a chemoattractant. Moreover, enhanced ALP activity and upregulated expression of osteogenic markers (RUNX2, COL, OPN) demonstrated the hydrogel’s dual angiogenic and osteogenic potential for bone tissue regeneration.

### Nanotubes

Nanotubes reinforce hydrogels through their high stiffness, strength, and aspect ratio, acting as rigid fillers that enhance composite stiffness and toughness. Their elongated structure enables efficient stress transfer and redistribution within the polymer network, reducing local deformation and improving durability. Strong interfacial interactions, such as hydrogen bonding, ionic, or covalent links, facilitate effective load transfer and act as additional crosslinking sites. At higher concentrations, nanotubes form interconnected networks that dissipate energy and bridge microcracks, further enhancing fracture resistance and mechanical stability.

### Carbon nanotubes

Carbon nanotubes (CNTs) are cylindrical nanostructures composed of carbon atoms arranged in a hexagonal lattice. They are classified by the number of graphene layers as single-walled carbon nanotubes (SWCNTs) or multi-walled carbon nanotubes (MWCNTs). CNTs possess exceptional electrical conductivity, thermal stability, and mechanical strength, making them attractive reinforcement materials for tissue engineering scaffolds [86].

CNTs enhance the mechanical properties of hydrogels through several mechanisms. Firstly, they are highly strong and stiff nanomaterials that, when incorporated into hydrogels, function as rigid fillers reinforcing the polymer network. Secondly, their elongated structure allows efficient stress transfer and even distribution of forces, minimizing localized deformation and improving the hydrogel’s toughness and overall mechanical integrity, which is advantageous for load-bearing applications [87]. Additionally, CNTs form an internal network within the hydrogel that dissipates stress, further enhancing resilience against mechanical deformation [88].

Functionalized CNTs can also establish noncovalent interactions with polymer chains, such as hydrogen bonding, which increase the effective crosslinking density of the hydrogel network and further improve its mechanical strength and stability [89].

Kaur *et al.* [90] formulated an injectable hydrogel composed of chitosan and collagen, reinforced with carboxylated SWCNTs (COOH-SWCNTs), and crosslinked with β-GP. Different concentrations of COOH-functionalized SWCNTs (COOH-SWCNTs) were sequentially added to the precursor and stirred for 24 h. The COOH groups improved CNT hydrophilicity and dispersibility, preventing aggregation and promoting uniform distribution within the chitosan/collagen hydrogel matrix. These functional groups also facilitated hydrogen bonding and possible covalent interactions with polymer chains, enhancing interfacial adhesion and stress transfer. Increasing COOH-SWCNT content (up to 5 wt.%) produced a more porous and aligned network, markedly increasing the Young’s modulus from the kilopascal range to values comparable with native bone, confirming improved stiffness and load-bearing capacity. The hydrogels were bioactive, forming a hydroxyapatite layer after 1 day in SBF, owing to the charged groups in CS, collagen, and COOH-SWCNTs that attracted Ca^2+^ and PO_4_^3-^ ions. Crosslinking via β-GP, combined with COOH-SWCNT integration, optimized thermoresponsive behaviour, injectability, and mechanical strength. These hydrogels were non-toxic and significantly enhanced cell proliferation and osteogenic differentiation compared to pure CS/collagen. Overall, the COOH-SWCNT-reinforced hydrogels represent mechanically robust, injectable biomaterials suitable for minimally invasive bone tissue engineering applications.

Liu *et al.* [91] developed a novel hydrogel system incorporating CNTs and black phosphorus (BP) within a biodegradable oligo(polyethylene glycol fumarate) (OPF) matrix. Acrylated polyethylene glycol was grafted onto carboxyl-functionalized CNTs to improve dispersion and surface compatibility. The resulting tubular CNT-PEG acrylate (CNTpega) introduced reactive acrylate groups, enabling covalent crosslinking within the hydrogel network and significantly enhancing mechanical performance, with compressive strength increasing from ~20 to ~160 kPa. The BP-CNTpega hydrogels supported cell adhesion, proliferation, focal adhesion formation, and osteogenic differentiation of pre-osteoblasts. Additionally, the inherent electrical conductivity of CNTs promoted osteogenesis under electrical stimulation by upregulating key osteogenic genes. *In vivo*, injectable BP-CNTpega hydrogels demonstrated rapid gelation, effectively filling bone defects in rabbit femur and vertebral models and facilitating posterolateral spinal fusion. Overall, these findings highlight the potential of this injectable hydrogel system for bone tissue engineering applications.

### Halloysite nanotubes

Halloysite nanotubes (HNTs) are naturally occurring aluminosilicate minerals with a hollow tubular morphology and the general formula Al_2_Si_2_O_5_(OH)_4_·nH_2_O. Due to their biodegradability, high mechanical integrity, and ability to carry therapeutic agents, HNTs have gained traction in various biomedical applications [92].

HNTs enhance the mechanical properties of hydrogels through several mechanisms. First, they act as rigid fillers within the polymer matrix due to their tubular structure and intrinsic stiffness [93]. The high aspect ratio and rigidity of HNTs facilitate efficient stress transfer from the soft polymer matrix to the nanotubes, reducing stress concentrations and preventing microcrack propagation, which enhances the overall fracture resistance and stiffness of the hydrogel [94]. Furthermore, hydroxyl groups on the HNT surface can form hydrogen bonds or other noncovalent interactions with polymer functional groups (-OH, -COOH, -NH_2_), improving interfacial adhesion and load transfer. These interactions serve as additional crosslinks that restrict polymer chain mobility, thereby increasing tensile and compressive strength and reducing swelling of the hydrogel network [93].

Kazemi-Aghdam *et al.* [95] modified HNTs with chitosan to fabricate mechanically enhanced injectable hydrogels for bone regeneration. The modified HNTs (mHNTs) were dispersed by stirring for 24 h. Incorporation of 8 wt.% mHNTs into a chitosan/β-GP thermoresponsive matrix increased the storage modulus from below 1 kPa to over 10 kPa. This enhancement was attributed to restricted polymer chain mobility, improved nanoparticle dispersion, strong interfacial interactions, and the intrinsic rigidity of HNTs. The addition of mHNTs also promoted the proliferation and osteogenic differentiation of encapsulated hASCs. The stiff, tubular structure of HNTs, combined with their ability to dynamically deliver icariin (IC) as an osteogenic inducer, further contributed to mechanical reinforcement and bioactivity. Overall, the IC@mHNTs-loaded thermosensitive hydrogel represents a promising injectable system for bone tissue engineering.

Maroufi *et al.* [96] designed nanocomposite hydrogels by blending chitosan, oxidized quince seed gum (OXQSG), and curcumin-loaded HNTs (CUR-HNTs). Different concentrations of CUR-HNTs were dispersed in the solution via continuous mechanical stirring for 24 h, followed by 20 min of sonication. The CUR-HNTs were predominantly cylindrical with minor agglomeration. Incorporation of 30 wt.% CUR-HNTs into the optimized chitosan/OXQSG matrix significantly enhanced mechanical performance, increasing ultimate strain from below 20 to ~30 %, indicating improved elasticity and toughness. This reinforcement is attributed to potential strain-induced alignment of the clay layers within the polymer matrix and hydrogen bonding interactions between the polymer and clay. Additionally, CUR-HNTs improved thermal stability, cell attachment, and proliferation. Overall, CS/OXQSG hydrogels at a 25:75 molar ratio with 30 wt % CUR-HNTs provide a mechanically robust and antibacterial scaffold suitable for tissue engineering applications.

### Nanofibers

Nanofibers reinforce hydrogels by enhancing network stiffness, load-bearing capacity, and overall mechanical integrity. They promote efficient stress transfer, reduce stress concentration, and impede crack propagation. Interactions between fibre surfaces and polymer chains, such as hydrogen bonding or ionic interactions, increase effective crosslinking density and network stability. When aligned or hierarchically organized, nanofibers provide directional reinforcement, further improving stiffness, strength, and toughness compared to randomly oriented fibres.

### Cellulose

Cellulose, a linear polysaccharide found in plant cell walls and various microorganisms, is widely utilized in biomedical applications due to its abundance, non-toxicity, biocompatibility, and cost-effectiveness [97]. Its nanostructured forms, such as cellulose nanofibers (CNFs) and cellulose nanocrystals (CNCs), provide enhanced surface area, mechanical strength, and biomimetic features favourable for tissue engineering [98]. Table 5 presents the outcomes of recent research on enhancing the mechanical properties of injectable hydrogels by utilizing cellulose.

**Table 5.** Mechanical properties reinforcement of injectable hydrogel based on cellulose NPs

Hydrogel composite	Form and content of cellulose used	Dispersion method / average size	Changes in mechanical properties	Ref.
Collagen, chitosan, gold NPs	Aldehyde modified nanocrystalline cellulose (ald-CNCs) used as the crosslinker	Sonication / spherical morphology with a ~50 to 70 nm mean diameter	Collagen/ald-CNCs with blending ratios of 1:2, 1:1, and 1:0.5 with tensile moduli of 0.51, 0.40 and 0.18 MPa	[102]
Chitosan	Physical blending of 0.5, 1 and 2 wt.% of CNCs	Sonication / Nanorods with length of 100 to 200 nm and diameter of 10 to 20 nm	Elastic modulus increases from 86 to 379 kPa by adding 2 wt.% of CNC	[103]
Poly (vinyl alcohol)	Physical blending of 0.5, 1 and 1.5 wt.% of nanocellulose fibrils	Ultrasonic bath / diameter of fibrils ranges from 20 to 50 nm	Storage modulus increases from 1 to 10 kPa by adding 1.5 wt % of nanocellulose fibrils	[104]
Chitosan, pectin	0.125 to 0.5 wt.% of ald-CNCs as crosslinkers, covalent Schiff base bonds with chitosan amino groups	Sonication / not given	Storage modulus increases from 0.1 to 1 kPa by adding 0.5% w of nanocellulose	[105]
Oxidized alginate, gelatine	Physical blending of 1, 3 and 5 wt.% of cellulose nanofibers (CNF)	Sonication / CNF with length of 1 to 3 μm and diameter of ~30 nm	Compressive strength rises from 51.3 to 102.6 KPa for CNF	[36]

Cellulose enhances the mechanical properties of hydrogels through several mechanisms. CNCs, owing to their high stiffness and aspect ratio, function as rigid nanofillers within hydrogel matrices [99]. The abundant surface hydroxyl (-OH) groups on CNCs facilitate hydrogen bonding and other noncovalent interactions with polymer chains, thereby enhancing interfacial adhesion and serving as additional physical crosslinks that stabilize the polymer network [100]. This reinforcement increases the effective crosslinking density and facilitates the formation of a percolated CNC network, which distributes stress more uniformly and dissipates energy under deformation, thereby improving the hydrogel’s toughness and crack resistance [101].

Maturavongsadit *et al.* [103] developed an injectable thermoresponsive hydrogel composed of chitosan and β-GP, reinforced with CNCs. Incorporation of 0.5 to 2 wt.% CNCs into the chitosan hydrogel markedly enhanced stiffness, increasing the elastic modulus from 28 kPa (plain hydrogel) to 379 kPa (2 wt.% CNCs), indicating strong interfacial interactions between CNCs and the polymer network. The CNC-CS hydrogels exhibited rapid gelation (<7 s) at 37 °C and maintained high cell viability (>80 %) for encapsulated pre-osteoblasts, which displayed well-developed cytoskeletons and branching morphologies. All formulations were biocompatible *in vivo* and showed significant biodegradation over 30 days. These results demonstrate that CNCs improve both the mechanical properties and gelling kinetics of chitosan hydrogels, supporting their potential as injectable, biodegradable scaffolds for cell delivery and bone tissue engineering. To our knowledge, this represents the first report of a chitosan-CNC hydrogel capable of delivering viable osteoblasts while promoting cell adhesion and differentiation.

Cui e*t al.* [36] synthesized a semi-interpenetrating network hydrogel using oxidized alginate (OSA), gelatine and varying concentrations of CNFs (1, 3 and 5 wt.%), without chemical crosslinkers. Compressive strength of the OSA/Gel/CNF hydrogels increased with CNF content, reaching 102.6 kPa at 5 wt.% CNF compared to 51.3 kPa for the control, demonstrating the reinforcing effect of CNFs. The hydrogels also exhibited excellent bioactivity, with cell viability exceeding 96 %, enhanced biomineralization and osteogenic activity (Ca/P molar ratio ≈1.69), indicating their strong potential for bone tissue repair applications.

### Silk

Silk fibroin (SF) protein, extracted from the cocoon of silkworms (Bombyx mori) and purified by removing sericin, exhibits exceptional biocompatibility, slow biodegradability, and remarkable mechanical properties. These features make SF a highly promising material for tissue engineering applications [106]. Moreover, SF provides suitable attachment sites for various cell types, facilitating cell adhesion and growth [107].

SF nanofibers enhance the mechanical properties of hydrogels through several mechanisms. Firstly, they contain β-sheet crystalline domains that provide high intrinsic stiffness [108]. When embedded in hydrogel matrices, these nanofibers act as reinforcing elements, bearing load, bridging microcracks, and enhancing the overall strength and durability of the network [109]. Secondly, when electrostatic repulsion between nanofibers is reduced, enabling their assembly into interconnected networks, stress can be more evenly distributed throughout the matrix. This interconnected nanofiber network facilitates efficient energy dissipation under mechanical loading, thereby improving the hydrogel’s toughness and resistance to deformation during repeated stress cycles [110].

Ding *et al.* [111] investigated the fabrication of silk nanofiber hydrogels loaded with desferrioxamine (DFO). The SF nanofibers had diameters ranging from 20 to 30 nm. Incorporation of DFO slightly enhanced the hydrogel’s modulus and viscosity, while adding 0.5 to 2 wt.% SF nanofibers significantly increased the storage modulus from below 1 kPa to over 10 kPa, without affecting shear-thinning behaviour. These hydrogels exhibited sustained zero-order release of DFO for more than 40 days *in vitro*, thereby reducing cytotoxicity and promoting vascularization. *In vivo*, DFO-loaded hydrogels improved angiogenesis, supported cell migration and function, and accelerated high-quality wound healing, demonstrating a versatile strategy for neovascularization and tissue repair.

In another study, Wang and colleagues developed DFO-loaded SF-HAp nanocomposite hydrogels by adsorbing DFO onto SF nanofibers and integrating hydroxyapatite (HAp) nanorods [112]. The nanocomposite hydrogels demonstrated markedly improved mechanical properties over pure SF nanofiber hydrogels, with the modulus exceeding 28 kPa, approximately a tenfold increase. This enhanced stiffness, reflecting the organic-inorganic structure of natural bone, indicates strong potential for promoting osteogenic differentiation.

### Ionic doping strategy

Recently, ionic doping of nanomaterials has been shown to not only enhance mechanical characteristics but also improve biological functions. Significant efforts have focused on incorporating various ionic substitutions, including Si^4+^, Mg^2+^, Zn^2+^ and CO_3_^2-^, into hydrogel scaffolds for bone regeneration. Among them, the divalent strontium ion (Sr^2+^) has attracted considerable attention due to its ionic radius (0.112 nm) being close to that of calcium (Ca^2+^, 0.10 nm) and its key physiological role in bone metabolism [113]. Even at trace concentrations, Sr^2+^ inhibits pre-osteoclast maturation and bone resorption while promoting osteoblast activity, collagen synthesis, and overall cell proliferation, thereby reducing bone loss and improving bone formation [114].

Strontium-based NPs (SrNPs) further enhance the potential of Sr^2+^ by offering increased bioavailability, sustained release, and tuneable surface features. Compared to conventional Sr salts, SrNPs can be engineered for targeted biological interactions, providing promising tools to combat bone diseases and facilitate tissue repair [115].

Sr-substituted nanowires provide enhanced mechanical reinforcement compared to spherical particles due to their high aspect ratio, which improves stress transfer, crack bridging, and load distribution within the hydrogel matrix. Controlled hydrothermal synthesis yields well-dispersed nanowires that form entangled networks, further strengthening the composite [116]. Substitution of Sr promotes changes in surface charge and ionic interactions, thereby strengthening bonding to polymer functional groups (-COO^-^, -OH) and enabling effective stress transfer. Additionally, the released Sr^2+^ ions act as ionic crosslinkers, inducing apatite-like mineralization and enhancing stiffness and long-term structural stability. The incorporation of Sr^2+^ into the HA or xonotlite lattice also alters crystallinity and lattice parameters, improving particle rigidity and interfacial compatibility with the polymer matrix [117,118].

Yu *et al*. [26] developed an injectable organic-inorganic nanocomposite hydrogel using gelatin methacryloyl (GelMA) and Sr-substituted xonotlite nanofibers. Ultra-long Sr-xonotlite nanowires (~20 nm in diameter and several micrometres in length) were dispersed by stirring. Their incorporation enhanced the hydrogel’s mechanical strength and promoted cell adhesion, proliferation, ALP activity, and the expression of osteogenic and angiogenic genes. *In vivo*, the composite hydrogel achieved significant bone regeneration at defect sites, demonstrating strong potential for in situ bone repair.

**Figure 5.**
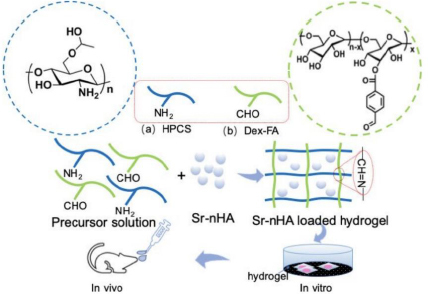
The *in situ* hydrogel was fabricated using HPCS and Dex-FA to prepare CDH hydrogel. The chemical structure of HPCS and Dex-FA was shown in the blue and green circle, respectively. For Sr-nHAp incorporation, different NPs were added into the Dex-FA solution and then mixed with HPCS solution. The results showed that Sr-nHAp NPs incorporation into CDH would significantly improve the rheological and mechanical properties as well as bone regeneration, compared to CDH without Sr-nHAp NPs [119] Copyright [2019] by American Chemical Society. Reprinted with permission

In another study, as illustrated in Figure 5, Ding *et al.* [119] aimed to enhance the mechanical and osteogenic performance of chitosan-based hydrogels by encapsulating SrNPs into the matrix.

Hydroxypropyl chitosan (HPCS) and dextran-4-formyl benzoic acid (Dex-FA) were crosslinked via imine bond formation to form hydroxypropyl chitosan/aldehyde dextran (CDH) hydrogels with a baseline compressive modulus of 21.65 ± 1 kPa. Incorporation of strontium-substituted nHAp (Sr-nHAp), prepared at various Sr/nHAp molar ratios and dispersed by sonication, markedly enhanced the mechanical and rheological performance. The controlled release of Sr^2+^ from Sr-nHAp/CDH further promoted osteoblast proliferation and osteogenic differentiation. This injectable, in situ-forming hydrogel demonstrates strong potential for bone regeneration in orthopedic, dental, and craniofacial applications.

### Nanosheet

Nanosheets enhance the mechanical properties of hydrogels through several synergistic mechanisms. Acting as rigid planar fillers, they increase stiffness and elastic modulus while providing extensive surface area for hydrogen bonding or ionic interactions with polymer chains, thereby serving as additional physical crosslinks. Their well-dispersed, layered structure enables uniform stress distribution, reduces localized deformation, and suppresses crack propagation. Moreover, the incorporation of nanosheets often increases the storage modulus and can modify gelation behaviour, yielding stiffer yet responsive hydrogel networks.

### Graphene oxide-based nanocomposite

Graphene oxide (GO), a two-dimensional carbon-based nanomaterial, has attracted significant interest in hydrogel reinforcement due to its unique physicochemical properties. Structurally, GO consists of a single atomic layer of carbon atoms, typically ~1 nm thick, with lateral dimensions that can reach several micrometres. Its high density of oxygen-containing functional groups-such as hydroxyl, epoxy, and carboxyl-confers excellent hydrophilicity, enabling stable dispersion in aqueous environments [120].

GO has demonstrated promising capabilities in enhancing the biological performance of composite materials, including improved drug loading, antimicrobial activity, and stimulation of cell proliferation. Although concerns regarding GO cytotoxicity exist, these effects are largely dose- and size-dependent. When incorporated into hydrogel systems, GO not only improves conventional properties such as mechanical strength and elasticity but also imparts functionalities like electrical conductivity and bioresponsiveness [121]. Table 6 summarizes recent studies on the mechanical reinforcement of injectable hydrogels using GO.

**Table 6.** Mechanical properties reinforcement of injectable hydrogel based on GO

Hydrogel omposite	Form and content of Sr used	Dispersion method / average size	Changes in mechanical properties	Ref.
Silk fibroin, carboxymethyl cellulose, agarose	Physical blending of 0, 0.1, 0.2 and 0.4 mg mL^-1^ of polydopamine functionalized GO (GO@PDA)	Stirred for 2 h / size of GO and GO@PDA were ⁓315±20 and 639±25 nm, respectively	At a strain of 60 % the stress reaches 60.2 kPa for 0.4 mg mL^-1^ of GO@PDA	[127]
Alginate, sericin	Physical blending of 10 to 100 μg ml^-1^ of GO	Not given / not given	Maximum compressive stress increased from 50 to 68 kPa with addition of 20 μg ml^-1^ of GO (0.1 ml)	[125]
Silk fibroin	Physical blending of 0.05, 0.1, 0.2 and 0.4 wt.% of GO	Sonication / not given	Compressive strength increased from 0.17 to 0.35 MPa by incorporating 0.4 wt.% of GO	[128]
Glycol chitosan, oxidized hyaluronic acid	Physical blending of 18, 36 and 72 μg ml^-1^ of GO	Solution blending and vortex-assisted dispersion / not given	Storage modulus increased by adding 18 to 72 μg ml^-1^ of GO from 3 kPa to 9 kPa	[126]
Chitosan methacrylate	Physical blending of 3 % w/v of GO	Sonication / not given	Storage modulus increased to 800 Pa by adding 3 % w/v of GO	[129]
Chitosan, poly N-isopropyl acrylamide	Chemical composition of 60 mg of GO with poly N-isopropyl acrylamide	Sonication / not given	Compressive strength increased from 0.4 to 9.7 MPa	[130]

GO enhances the mechanical properties of hydrogels through several mechanisms. First, the incorporation of GO acts as a rigid filler, enhancing the strength and elastic modulus of hydrogels [122]. In addition, strong interfacial interactions, such as hydrogen bonding or ionic interactions between the functional groups of GO and polymer chains, increase the effective crosslinking density within the network. In some systems, GO also serves as a crosslinking agent, further improving mechanical stability and introducing functional properties such as self-healing behaviour [123]. Moreover, GO reduces hydrogel swelling by restricting water mobility and maintaining a more compact network structure [124].

Jiang *et al.* [125] developed an injectable alginate-tyramine hydrogel that was crosslinked enzymatically using horseradish peroxidase (HRP) and hydrogen peroxide and reinforced with sericin and GO nanosheets. The alginate/sericin/GO hydrogel demonstrated controlled degradation and inherent bioimaging capability. Incorporation of GO enhanced rBMSC spreading, osteogenic differentiation, and mineralization. The formulation containing 20 μg mL^-1^ GO achieved the highest compressive strength (68 kPa at 36 % strain) and compression modulus (16.5 kPa), with excellent strain recovery under cyclic loading. Sericin reduced early inflammation and promoted M2 macrophage polarization, supporting BMSC differentiation, while GO further stimulated osteogenesis. Together, sericin and GO synergistically improved bone regeneration, yielding an injectable, traceable, and biocompatible hydrogel for bone repair.

In another study, Lee *et al.* [126] fabricated a GO-incorporated injectable hydrogel using glycol chitosan and oxidized hyaluronic acid (HA). Increasing GO content from 18 to 72 μg mL.^-1^ progressively raised the hydrogel’s storage modulus from below 3 kPa to over 9 kPa. This improvement is attributed to GO forming bridging domains within the polymer matrix *via* strong hydrogen bonding and interfacial interactions, reinforcing the network. The GO-incorporated injectable hydrogel exhibited minimal toxicity while promoting excellent osteogenic activity, as confirmed *in vitro* and *in vivo*. These results highlight its potential as a mechanically robust, osteoinductive hydrogel for bone tissue engineering applications.

## Future perspectives

Each class of nanoparticle provides distinct structural and functional benefits for injectable hydrogels; however, several scientific and translational challenges remain. Hydroxyapatite NPs, though widely used for bone regeneration due to their compositional similarity to native bone, still face limitations in mechanical robustness and degradation stability under high-stress conditions compared with emerging materials such as graphene and bioactive glass [131]. Similarly, bioactive glass, whitlockite, and calcium phosphate NPs provide strong mechanical reinforcement but are prone to brittleness, particle aggregation, and uncontrolled ion release, which can disrupt scaffold degradation rates and induce local cytotoxicity [132].

Nanotubes provide excellent mechanical and electrical reinforcement but raise persistent concerns regarding long-term biocompatibility and chronic toxicity. Depending on their size and morphology, carbon nanotubes may accumulate in organs such as the liver, spleen, and lungs, potentially eliciting inflammatory or carcinogenic responses [133]. Nanofibers, particularly cellulose nanofibers (CNFs), are attractive due to their renewability, flexibility, and tuneable stiffness, yet issues such as excessive viscosity, reliance on toxic crosslinkers, and complex purification hinder their large-scale and safe biomedical use [134]. Graphene-based materials, including graphene oxide (GO), also pose potential risks of cellular toxicity, despite their outstanding reinforcement and conductivity properties [135].

To translate these advances into clinical practice, future efforts should focus on designing minimally invasive, injectable nanocomposite hydrogels with predictable degradation profiles and high bioactivity. Incorporating bioadhesive or self-healing mechanisms could improve fixation at defect sites and eliminate the need for additional stabilization materials. Progress in scalable manufacturing, standardized synthesis protocols, and rigorous regulatory validation will also be critical for ensuring safety, reproducibility, and clinical reliability [136,137].

A key limitation of existing studies, such as those involving whitlockite-based systems, is methodological heterogeneity. Variations in experimental design, animal models, and outcome measures impede direct comparison and meta-analysis, while the predominant reliance on small-animal, non-load-bearing models restricts clinical extrapolation [138].

Emerging directions include AI-driven materials design, multiphase nanocomposites, and stimuli-responsive hydrogels capable of adaptive remodelling *in vivo* [139-141]. Integrating machine learning and data-informed modelling can accelerate the optimization of nanoparticle-hydrogel interactions, enabling multifunctional systems that promote osteogenesis, angiogenesis, and controlled degradation simultaneously. Given the growing availability of materials databases and computational tools, AI-assisted hydrogel engineering represents a transformative step toward the rational design of next-generation biomaterials for bone tissue regeneration [142-144].

## Conclusions

Recent developments in bone tissue engineering have underscored the potential of injectable hydrogels as minimally invasive and adaptable scaffolds that can fill irregular defects and support cellular interactions within the extracellular matrix. However, the limited mechanical strength of conventional hydrogels remains a key obstacle compared to native bone. Incorporating nanomaterials has proven effective for reinforcing these systems, significantly improving stiffness, compressive strength, and biological performance.

Despite these advances, nanoparticle-reinforced injectable hydrogels still face challenges such as brittleness, uncontrolled ion release, cytotoxicity, and the lack of standardized fabrication and clinical validation. Furthermore, the absence of consistent *in vivo* load-bearing models hinders direct comparison and clinical translation.

Future research should aim to develop multifunctional, bioadhesive, and injectable hydrogel systems capable of promoting osteogenesis, angiogenesis, and controlled degradation. The integration of AI-driven material design, multiphase nanocomposites, and stimuli-responsive networks offers a promising route toward optimizing mechanical and biological performance. Ultimately, continued interdisciplinary collaboration will be essential to translate these advanced nanocomposite hydrogels into clinically viable platforms for effective bone regeneration.
